# Only, O Man

**Published:** 1889-06

**Authors:** Thomas L. Harris


### ONLY, O MAN.

Only, O man, as thou dost cease
Thy civic feuds, and live in peace,
And give unto the poor release;
Only as thou abjurest self,
Lovest thy brother more than pelf,
And drivest out the impish elf,
Sectarian pride, from all thy heart,
Canst thon have place, or lot, or part
Within the Heaven-created mart
Of angel love and angel bliss;
And when thy bosom findeth this,
Thy lips shall feel the Spirit's kiss.

—THOMAS L. HARRIS.

